# Novel Integrated Tiered Cumulative Risk Assessment of Heavy Metals in Food Homologous Traditional Chinese Medicine Based on a Real-Life-Exposure Scenario

**DOI:** 10.3389/fphar.2022.908986

**Published:** 2022-06-24

**Authors:** Tian-Tian Zuo, Hong-Yu Jin, An-Zhen Chen, Lei Zhang, Shuai Kang, An-Ping Li, Fei Gao, Feng Wei, Jian-Dong Yu, Qi Wang, Jian-Bo Yang, Shuang-Cheng Ma

**Affiliations:** ^1^ National Institutes for Food and Drug Control, Beijing, China; ^2^ Qingdao Institute for Food and Drug Control, Qingdao, China; ^3^ China National Center for Food Safety Risk Assessment, Beijing, China; ^4^ Gansu Institute of Drug Control, Lanzhou, China

**Keywords:** traditional Chinese medicine (TCM), food homologous TCM, heavy metals, co-exposure, cumulative risk assessment

## Abstract

In order to serve population health better, the first integrated tiered decision tree for cumulative risk assessment of co-exposure of Pb-, Cd-, and As-associated health risks in food homologous traditional Chinese medicine (TCM) was designed, after measuring their concentrations by inductively coupled plasma-mass spectroscopy (ICP-MS). Basically, our three-step decision tree involving hazard quotient (HQ), hazard index (HI), and target-organ toxicity dose (TTD) modification of the HI method was developed to evaluate the potential risks of 949 batches of 15 types of food homologous TCM. To acquire a real-life exposure scenario, the cumulative risk assessment model was established by optimizing key parameters, such as ingestion rates, frequency, and duration of exposure to food homologous TCM based on questionnaire data. As a result, the mean concentrations of Pb, Cd, and As in 949 batches of food homologous TCM were 0.896, 0.133, and 0.192 mg/kg, respectively. The HQ values of As for *Angelica sinensis* (Oliv.) Diels and *Houttuynia cordata* Thunb. were 1.04 and 1.01, respectively, for females. Other HQs of Pb, Cd, or As in food homologous TCM were lower than 1 for both males and females. However, after rapid screening of the co-exposure health risks of heavy metals by the HI method, cumulative risk assessment results acquired by TTD modification of the HI method implied that the potential health risks associated with the co-exposure of Pb, Cd, and As in *Lonicera japonica* Thunb. and *Houttuynia cordata* Thunb. ingested as both TCM and food were of concern in the clinic. Additionally, the cumulative risks of Pb, Cd, and As in *Mentha canadensis* L., *Chrysanthemum indicum* L., and *Zaocys dhumnades* (Cantor) only used as food exceeded the human tolerance dose. Collectively, our innovation on the tiered strategy of decision tree based on a real-life exposure scenario provides a novel approach engaging in the cumulative risk assessment of heavy metals in food homologous TCM. All in all, such effort attempts to scientifically guide the rational use of TCM in the treatment of the complex diseases and the improvement of population health.

## 1 Introduction

Currently, global focus has been directed towards heavy metals due to their damaging effects on human health (Bortey-Sam et al., 2015; Gündüz, 2015; Kashyap et al., 2018). Heavy-metal exposure and pollution is a severe problem because they are abundant in the environment, non-biodegradable, persistent, and highly toxic even at trace levels (Auyeung et al., 2002; Arjouni et al., 2015; Emenike et al., 2017; Maghakyan et al., 2017; Jiang et al., 2018), and they exert deleterious effects likely by disturbing the cell metabolism, ionic transportation, protein folding, and DNA modification (Sirot et al., 2009; Jaishankar et al., 2014). Pb is one of the most poisonous metals; chronic exposure to Pb affects the normal functioning of the nervous system, cardiovascular system, reproductive system, and kidneys, leading to reduced consciousness, increased blood pressure, loss of appetite, hyperactivity, anemia, and fatigue (Auyeung et al., 2002; Bellinger, 2008; Buettner et al., 2009; Barakat, 2011; Bolan et al., 2016; Bassil et al., 2018). Excessive exposure to Cd leads to a decline in cognitive capacity, reproductive deficiencies, kidney dysfunction, osteoporosis, fractures, and diabetes (Chen et al., 2014; Zhang et al., 2014; Sanders et al., 2015). Inorganic As has been recognized as a group I carcinogen without a threshold and could increase the risk of kidney dysfunction, excessive stillbirth, and immune system diseases (Hughes, 2002; Kapaj et al., 2006).

Food homologous traditional Chinese medicine (TCM) is more frequently and commonly used TCM in daily life and more closely related to human health. There is a long history of food homologous TCM usage, which has been well described in various studies on traditional medicine, including “The Book of Poetry” since XiZhou Dynasty, “Shen Nong’s Herbal Classic of Materia Medica” 1800 years ago, and “Compendium of Materia Medica” in the 16th century (Qiu, 2007; Stone, 2008; Song et al., 2020). The “Compendium of Materia Medica” recorded 25 varieties of food homologous TCM, and more than 100 varieties have been included in the list of food homologous TCM launched by the National Health Commission of People’s Republic of China in 2020. Owing to its excellent performance and satisfactory health effects, food homologous TCM is used extensively and has received worldwide recognition in modern society (Cao et al., 2014; Yue et al., 2017). In addition, food homologous TCM is a popular raw material for food supplements as well as complementary medicine and has been introduced in other Asian and developed countries. Over the past two decades, the global consumption of food supplements has increased dramatically, with an estimated $33.9, $1.31, and $7.18 billion spent on food supplements annually in the United States, England, and Canada, respectively (WHO, 2005; CMA, 2012). Excitingly, it has been reported recently that some food homologous TCM, such as *Lonicera japonica* Thunb. and *Chrysanthemum indicum* L., could be used to prevent SARS-CoV-2 (Song et al., 2020). However, food homologous TCM may be contaminated with heavy metals during cultivation, harvesting, and processing (Chan, 2003; Nagarajan et al., 2014). The consumption of food homologous TCM polluted by heavy metals through food chains causes the accumulation of these harmful environmental pollutants in humans and may pose severe public health risks. Therefore, it is crucial to scientifically assess the health risks of heavy metals in food homologous TCM and guide their safe use.

Common risk assessment methodologies for assessing risks posed by heavy metals are typically based on exposure to a single chemical, which is of significance for preliminary monitoring of the risk of a certain pollutant. However, this approach creates problems, involving underestimating the risks and lacking information about the full scope of health impacts due to co-exposure of multiple heavy metals. Actually, humans are exposed to numerous pollutants through a variety of pathways in real life (Akbari et al., 2012; Tsakiris et al., 2015; Bopp et al., 2018). In 1997, the World Health Organization (WHO) emphasized that simultaneous exposure to different chemicals should receive serious attention in food safety assessments, especially when such data are evaluated for regulatory purposes (WHO, 1997). The co-exposure caused by multiple pollutants *via* different pathways and media is called cumulative exposure, and the assessment of health risk caused by cumulative exposure is called cumulative risk assessment. This confronts experts and scientists with challenging tasks regarding the toxicological effects of contaminant mixtures and appropriate risk assessment approaches (Council, 1993). Substantial efforts have been made to explore the cumulative risk assessment methodologies, and recommendations from public organizations and authorities, involving the WHO/International Programme on Chemical Safety, European Commission non-food Scientific Committees, and the United States Environmental Protection Agency (US EPA), are available in the field of food and environment EPA, 2000; ATSRD, 2004a; ATSRD, 2004b; Meek et al., 2011; Kienzler et al., 2016). However, to the best of our knowledge, limited data about appropriate methods for cumulative risk assessment of TCM or food homologous TCM are available.

Given the globalization and popularity of food homologous TCM as well as the research gap in cumulative risk assessment strategy for TCM, the hypothesis of this study is that a newly cumulative risk assessment strategy for food homologous TCM is urgently needed. Doing so would allow one to scientifically evaluate the health risks of heavy metals based on a real-life exposure scenario. Thus, the main aims of the present study were: 1) to monitor the levels of Pb, Cd, and As in 949 batches of 15 types of food homologous TCM; 2) to assess the human health risks associated with each metal by hazard quotient (HQ); 3) to explore and perform the first cumulative risk assessment with regard to co-exposure of Pb, Cd, and As in food homologous TCM by hazard index (HI) and target-organ toxicity dose (TTD) modification of HI method; and 4) to exploit and figure out the first tiered strategy of a cumulative risk assessment for food homologous TCM.

## 2 Materials and Methods

### 2.1 Sample Collection

Representative and commonly used food homologous TCM samples were collected (Table 1) from TCM markets or retail pharmacies. A total of 949 batches of 15 types of food homologous TCM were collected from different environmental areas in China (between 86°27′E and 130°12′E and 20°02′N to 46°57′N). The samples were authenticated by Shuai Kang, an associated researcher on the identification of both herbal and animal medicinal materials.

**TABLE 1:** Sample collection information in this study.

Type	Category	Location
*Mentha canadensis* L. [Lamiaceae; *Menthae haplocalycis* herba]	Leaf	Hebei, Yunnan, Jilin, Anhui, Henan, Hubei, Guangxi, Shandong, Jiangsu, Jiangxi, Sichuan
*Ziziphus mauritiana* Lam. [Rhamnaceae; *Mauritianae* fructus]	Fruit	Shanxi, Shaanxi, Henan, Hebei, Xinjiang
*Angelica sinensis* (Oliv.) Diels [Apiaceae; *Angelica sinensis* radix]	Radix and rhizome	Gansu, Hubei
*Codonopsis pilosula* (Franch.) Nannf. [Campanulaceae; *Codonopsis* radix]	Radix and rhizome	Gansu, Heilongjiang
*Lycium barbarum* L. [Solanaceae; *Lycii* fructus]	Fruit	Xinjiang, Ningxia, Qinghai, Hebei, Beijing
*Lonicera japonica* Thunb. [Caprifoliaceae; *Lonicerae japonicae* flos]	Flower	Henan, Shandong, Hebei, Hubei, Anhui
*Prunus armeniaca* L. [Rosaceae; *Armeniacae semen* amarum]	Seed	Gansu, Hebei, Shanxi, Inner Mongolia, Ningxia
*Panax ginseng* C.A. Mey. [Araliaceae; *Ginseng* radix et rhizoma]	Radix and rhizome	Jilin, Heilongjiang
*Perilla frutescens* (L.) Britton [Lamiaceae; *Perillae* fructus]	Fruit	Hubei, Sichuan, Jiangsu, Henan, Anhui, Guangxi, Guangdong, Gansu, Hubei, Henan, Shanxi, North Korea
*Ziziphus jujuba* Mill. [Rhamnaceae; *Jujubae* fructus]	Seed	Shandong, Shanxi, Henan, Hebei, Liaoning
*Chrysanthemum indicum* L. [Asteraceae; *Chrysanthemi* flos]	Flower	Anhui, Hebei, Shandong, Henan, Jiangsu, Sichuan, Zhejiang, Hubei
*Chaenomeles lagenaria* (Loisel.) Koidz.[Rosaceae; *Chaenomelis* fructus]	Fruit	Yunnan, Hubei, Anhui, Sichuan, Henan
*Polygonatum odoratum* (Mill.) Druce [Asparagaceae; *Polygonati odorati* rhizoma]	Radix and rhizome	Zhejiang, Hunan
*Houttuynia cordata* Thunb. [Saururaceae; Houttuyniae herba]	Herb	Hubei, Sichuan, Jiangxi, Guangxi
*Zaocys dhumnades* (Cantor) [Colubridae; Zaocys]	Animal	Zhejiang,Hubei, Sichuan, Jiangxi

### 2.2 Sample Preparation, Analysis, and Quality Assurance

0.5 g homogenous powder of samples was placed into a microwave digestion system for digestion with 8.0 ml of HNO_3_. Then Pb, Cd, and As contents in the food homologous TCM samples were determined using an Agilent 7700X inductively coupled plasma-mass spectroscopy (ICP-MS, Agilent 7700X, Agilent Technologies Co., United States). For quality assurance, blanks and duplicates were analyzed throughout the analysis process. The internal standard was used to compensate for signal drift and matrix effects, and the mean recovery rates of the internal standard solution ranged from 92.9% to 108.3%. To guarantee the accuracy of the method, the mean percentage recovery rates of Pb, Cd, and As in *Angelica sinensis* (Oliv.) Diels (*n* = 9) were determined, which was 91% ± 0.8%, 93% ± 0.6%, and 104.3% ± 0.3%, respectively.

### 2.3 Health Risk Assessment

#### 2.3.1 Health Risk Assessment Based on a Single Chemical

To assess the health risks owing to ingestion of a single poisonous element in food homologous TCM, estimated daily intake (EDI) (μg/kg bw/day) and HQ were calculated using the following equations (Singh et al., 2010; Roba et al., 2016; Zuo et al., 2020a):

$$\text{EDI}=\frac{\text{EF}\times \text{Ed}\times \text{IR}\times \text{C}\times \text{t}}{\text{AT}\times \text{W}}.$$ (1)

Considering the unique characteristics of food homologous TCM used as both medicine and food, the risk assessment was conducted in two circumstances. In the first scenario, the food homologous TCM were used as TCM. Based on our 20,917 face-to face questionnaires obtained from 9,420 male volunteers and 11,497 female volunteers, the exposure frequency (EF), the exposure duration (Ed), and the mean value of daily ingestion rate (IR) for TCM was 90 days/year, 20 years, and 200 g/d, respectively (Zuo et al., 2020b); the concentration (C) of Pb, Cd, or As was detected in samples by ICP-MS (mg/kg); According to our previous study, the transfer rates of heavy metals are <10% for most TCMs^40^. Herein, the transfer rate used was assumed to be 10%; AT represented the average exposure time to TCM, which was equal to 365 d/y × 70 y; W was the average body mass. Based on the latest statistical data presented by the National Health Commission of the People’s Republic of China in 2020, the average adult body weights of males and females were 69.6 and 59 kg, respectively.

In the other scenario, where food homologous TCM were used as food, the P95 value of EF was 260 d/y and IR was 500 g/d, according to our questionnaire data. Ed was assumed to be 70 years, the average lifetime. Other parameters in the equation were consistent with those when food homologous TCM were used as TCM.

$$\text{HQ}=\frac{\text{EDI}\times \text{SF}\times 0.001}{\text{RfD}},$$ (2)

where RfD is the oral reference dose. The recommended values for Pb, Cd, and As were 0.0035, 0.001, and 0.0003 mg/kg bw/day, respectively (Mahmood and Malik, 2013). SF was the safety factor for food homologous TCM. Based on guidelines by the National Science Foundation (NSF), a 10% allocation of RfD was included to account for the contribution of dietary supplements as a component of daily food intake (International, 2003). In this case, the risks to human health would not be significantly increased. Thus, SF was 10 and 1 for TCM and food, respectively. Based on the Agency for Toxic Substances and Disease Registry (ATSRD)’s guidance manual, if at least two chemicals have HQs > 0.1, further assessment of additivity and interactions are necessary (ATSRD, 2001).

### 2.4 Preliminary Cumulative Risk Assessment Based on the Hazard Index Method

Joint toxic action may increase the hazard of a chemical mixture above that predicted by the assessment of individual components; therefore, the cumulative adverse effects raised by toxic metals in food homologous TCM should be considered. Exposure to more than one hazardous pollutant may result in additive effects and preliminary cumulative risk assessment was performed using the classic hazard index (HI) methodology, based on the following equation (Zuo et al., 2020a):

$$\text{HI}\;=\;{\text{HQ}}_{\text{Pb}}+{\text{HQ}}_{\text{Cd}}+{\text{HQ}}_{\text{As}}\text{.}$$ (3)

### 2.5 Precise Cumulative Risk Assessment Based on Target-Organ Toxicity Dose Modification of the Hazard Index Method

The TTD modification of the HI method was developed for the assessment accommodation of mixtures whose components do not have the same critical effects but have overlapping organs of toxicity. For the TTD modification of HI method, the toxicological effects of each pollutant (Pb, Cd, or As) based on each specific target organ were considered. Chemicals with multiple target organs have multiple target-organ toxicity doses, which were expressed as specific health-based guidance values (SHBGV) for assessing joint toxic actions of Pb, Cd, and As in food homologous TCM, and specific-end-point HQs were calculated based on Eq. 2 to obtain the TTD-modified HI values. According to the guidelines issued by ATSDR, the SHBGV of Pb for cardiovascular, hematological, neurological, renal, and testicular systems were 0.0013, 0.0042, 0.0042, 0.0006, and 0.0167 mg/kg bw/day, respectively. The SHBGV of Cd for cardiovascular, hematological, neurological, renal, and testicular systems were 0.005, 0.0008, 0.0002, 0.00083, and 0.003 mg/kg bw/day, respectively. Further, the SHBGV of As for cardiovascular, hematological, neurological, and renal systems were 0.0003, 0.0006, 0.0003, and 0.09 mg/kg bw/day, respectively (2004b).

### 2.6 Statistical Analysis

Statistical analysis was carried out using SPSS 22.0 (IBM Corporation, Armonk, NY, United States). Figures were plotted using GraphPad 6.0 software (San Diego, CA, United States).

## 3 Results

### 3.1 Pb, Cd, and As Determined by Inductively Coupled Plasma-Mass Spectroscopy in Food Homologous Traditional Chinese Medicine

The permissible maximum limits in the 2020 edition of the Chinese Pharmacopeia for Pb, Cd, and As in herbal medicine are 5, 1, and 2 mg/kg, respectively. The levels of Pb, Cd, and As varied among types of food homologous TCM, as shown in Table 2. Generally, the mean concentrations of Pb, Cd, and As in 949 batches of food homologous TCM were 0.896, 0.133, and 0.192 mg/kg, respectively. The mean levels of heavy metals in the 15 types of analyzed food homologous TCM varied from 0.024 to 3.126 mg/kg for Pb, 0.002 to 0.656 mg/kg for Cd, and 0.011 to 0.517 mg/kg for As. The mean levels revealed that among the 15 types of food homologous TCM, *Lonicera japonica* Thunb., *Houttuynia cordata* Thunb., and *Angelica sinensis* (Oliv.) Diels were the highest accumulators of Pb, Cd, and As, respectively. However, the contents of Pb, Cd, and As in *Ziziphus mauritiana* Lam. were lower than other food homologous TCM.

**TABLE 2:** Average content of Pb and Cd in MFHV.

Type	Batch no.	Content of heavy metals (mg/kg)		
		Pb	Cd	As
*Mentha canadensis* L.	81	1.836	0.116	0.329
*Ziziphus mauritiana* Lam.	71	0.024	0.002	0.011
*Angelica sinensis* (Oliv.) Diels	44	0.512	0.024	0.517
*Codonopsis pilosula* (Franch.) Nann	132	0.313	0.046	0.305
*Lycium barbarum* L.	62	0.211	0.075	0.070
*Lonicera japonica* Thunb.	74	3.126	0.284	0.208
*Prunus armeniaca* L.	62	0.053	0.006	0.017
*Panax ginseng* C.A. Mey.	50	0.443	0.076	0.054
*Perilla frutescens*(L.) Britton	61	0.787	0.126	0.213
*Ziziphus jujuba* Mill.	63	0.332	0.027	0.12
*Chrysanthemum indicum* L.	79	0.888	0.257	0.270
*Chaenomeles lagenaria* (Loisel.) Koidz.	33	0.181	0.037	0.099
*Polygonatum odoratum* (Mill.) Druce	61	0.585	0.213	0.067
*Houttuynia cordata* Thunb.	60	2.641	0.656	0.502
*Zaocys dhumnades* (Cantor)	16	1.508	0.045	0.197

### 3.2 Health Risk Assessment

**FIGURE 1:**
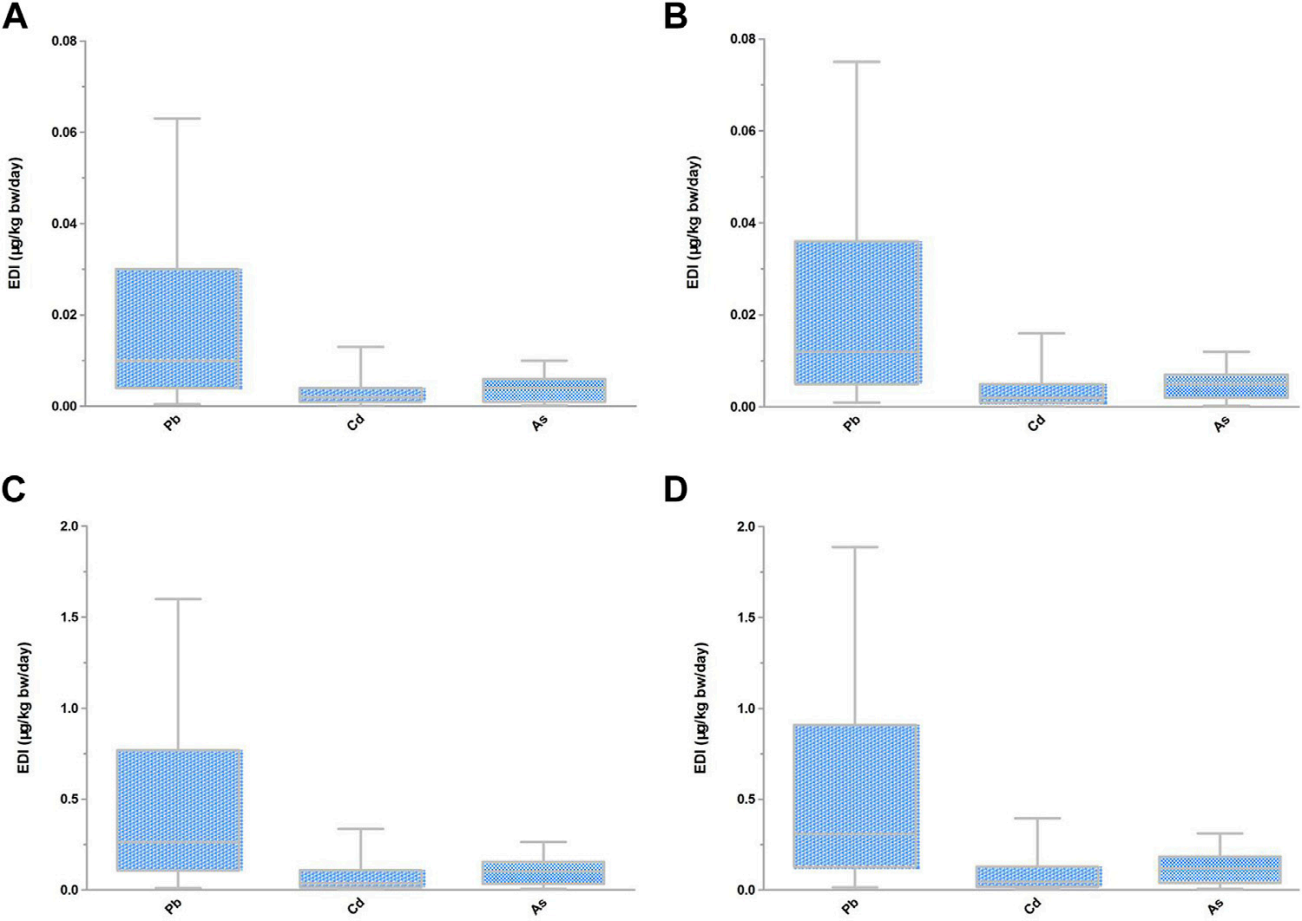
EDI of heavy metals in food homologous TCM. **(A)** EDI of heavy metals in food homologous TCM used as TCM for male. **(B)** EDI of heavy metals in food homologous TCM used as TCM for female. **(C)** EDI of heavy metals in food homologous TCM used as food for male. **(D)** EDI of heavy metals in food homologous TCM used as food for female.

The EDI values revealed that the highest intakes of Pb, Cd, and As occurred with the ingestion of *Lonicera japonica* Thunb., *Houttuynia cordata* Thunb., and *Angelica sinensis* (Oliv.) Diels, respectively (Table 3). For circumstances in which food homologous TCM were used as TCM, the EDI of Pb, Cd, and As ranged from 4.8 × 10^−4^ to 0.063 μg/kg/d, 4.0 × 10^−5^ to 0.013 μg/kg/d, and 2.2 × 10^−4^ to 0.010 μg/kg/d, respectively, for males; and from 0.001 to 0.075, 4.8 × 10^−5^ to 0.016 μg/kg/d, and 2.6 × 10^−4^ to 0.012 μg/kg/d, respectively, for females. For circumstances in which food homologous TCM were used as food, the EDI of Pb, Cd, and As ranged from 0.012 to 1.599 μg/kg/d, 0.001–0.336 μg/kg/d, and 0.006–0.264 μg/kg/d, respectively, for males; and from 0.014 to 1.887 μg/kg/d, 0.001–0.396 μg/kg/d, and 0.007 to 0.312, respectively, for females (Figure 1).

**TABLE 3:** EDI (μg/kg/d) and HQ for heavy metals due to consumption of MFHV used as TCM and food, respectively.

Types			TCM			Food		
			Pb	Cd	As	Pb	Cd	As
*Mentha canadensis* L.	Male	EDI	0.037	0.002	0.007	0.939	0.059	0.168
		HQ	0.105	0.023	0.219	0.268	0.059	0.561
	Female	EDI	0.044	0.003	0.008	1.108	0.070	0.199
		HQ	0.126	0.028	0.263	0.317	0.070	0.662
*Ziziphus mauritiana* Lam.	Male	EDI	4.80 × 10^−4^	4.00 × 10^−5^	2.20 × 10^−4^	0.012	0.001	0.006
		HQ	0.001	0.001	0.007	0.004	0.001	0.019
	Female	EDI	0.001	4.80 × 10^−5^	2.64 × 10^−4^	0.014	0.001	0.007
		HQ	0.002	0.001	0.009	0.004	0.001	0.022
*Angelica sinensis* (Oliv.) Diels	Male	EDI	0.010	4.80 × 10^−4^	0.010	0.262	0.012	0.264
		HQ	0.029	0.005	0.345	0.075	0.012	0.881
	Female	EDI	0.012	0.001	0.012	0.309	0.014	0.312
		HQ	0.035	0.006	0.414	0.088	0.014	1.040
*Codonopsis pilosula* (Franch.) Nannf	Male	EDI	0.006	0.001	0.006	0.160	0.024	0.156
		HQ	0.018	0.009	0.203	0.046	0.024	0.520
	Female	EDI	0.008	0.001	0.007	0.189	0.028	0.184
		HQ	0.021	0.011	0.244	0.054	0.028	0.614
*Lycium barbarum* L.	Male	EDI	0.004	0.002	0.001	0.108	0.038	0.036
		HQ	0.012	0.015	0.047	0.031	0.038	0.119
	Female	EDI	0.005	0.002	0.002	0.127	0.045	0.042
		HQ	0.014	0.018	0.056	0.036	0.045	0.141
*Lonicera japonica* Thunb.	Male	EDI	0.063	0.006	0.004	1.599	0.145	0.106
		HQ	0.179	0.057	0.139	0.457	0.145	0.355
	Female	EDI	0.075	0.007	0.005	1.887	0.171	0.126
		HQ	0.214	0.068	0.166	0.539	0.171	0.418
*Prunus armeniaca* L.	Male	EDI	0.001	1.20 × 10^−4^	3.40 × 10^−4^	0.027	0.003	0.009
		HQ	0.003	0.001	0.011	0.008	0.003	0.029
	Female	EDI	0.001	1.44 × 10^−4^	4.08 × 10^−4^	0.032	0.004	0.010
		HQ	0.004	0.001	0.014	0.009	0.004	0.034
*Panax ginseng* C.A. Mey.	Male	EDI	0.009	0.002	0.001	0.227	0.039	0.028
		HQ	0.025	0.015	0.036	0.065	0.039	0.092
	Female	EDI	0.011	0.002	0.001	0.267	0.046	0.033
		HQ	0.025	0.015	0.036	0.076	0.046	0.109
*Perilla frutescens* (L.) Britton	Male	EDI	0.016	0.003	0.004	0.403	0.064	0.109
		HQ	0.045	0.025	0.142	0.115	0.064	0.363
	Female	EDI	0.019	0.003	0.005	0.475	0.076	0.129
		HQ	0.054	0.030	0.170	0.136	0.076	0.428
*Ziziphus jujuba* Mill.	Male	EDI	0.007	0.001	0.002	0.170	0.014	0.061
		HQ	0.019	0.005	0.080	0.049	0.014	0.205
	Female	EDI	0.008	0.001	0.003	0.200	0.016	0.072
		HQ	0.023	0.006	0.096	0.057	0.016	0.241
*Chrysanthemum indicum* L.	Male	EDI	0.018	0.005	0.005	0.454	0.131	0.138
		HQ	0.051	0.051	0.180	0.130	0.131	0.460
	Female	EDI	0.021	0.006	0.006	0.536	0.155	0.163
		HQ	0.061	0.062	0.216	0.153	0.155	0.543
*Chaenomeles lagenaria* (Loisel.) Koidz.	Male	EDI	0.004	0.001	0.002	0.093	0.019	0.051
		HQ	0.010	0.007	0.066	0.026	0.019	0.169
	Female	EDI	0.004	0.001	0.002	0.109	0.022	0.060
		HQ	0.012	0.009	0.079	0.031	0.022	0.199
*Polygonatum odoratum* (Mill.) Druce	Male	EDI	0.012	0.004	0.001	0.299	0.109	0.034
		HQ	0.033	0.043	0.045	0.085	0.109	0.114
	Female	EDI	0.014	0.005	0.002	0.353	0.129	0.040
		HQ	0.040	0.051	0.054	0.101	0.129	0.135
*Houttuynia cordata* Thunb.	Male	EDI	0.053	0.013	0.010	1.351	0.336	0.257
		HQ	0.151	0.131	0.335	0.386	0.336	0.856
	Female	EDI	0.063	0.016	0.012	1.594	0.396	0.303
		HQ	0.181	0.157	0.402	0.455	0.396	1.010
*Zaocys dhumnades* (Cantor)	Male	EDI	0.030	0.001	0.004	0.771	0.023	0.101
		HQ	0.086	0.009	0.131	0.220	0.023	0.336
	Female	EDI	0.036	0.001	0.005	0.910	0.027	0.119
		HQ	0.103	0.011	0.158	0.260	0.027	0.396

### 3.3 Health Risk Assessment Based on a Single Metal

To assess the health risk associated with chronic exposure to heavy metals in diverse food homologous TCM, the HQ for Pb, Cd, and As was determined (Table 3). All HQ values of Pb, Cd, and As for the 15 types of food homologous TCM used as TCM were lower than 1 for both males and females. When the analyzed food homologous TCM were used as food, the HQ values of As for *Angelica sinensis* (Oliv.) Diels and *Houttuynia cordata* Thunb. were 1.04 and 1.01, for females, respectively. Other HQs of Pb, Cd, or As in food homologous TCM were lower than 1 for both males and females.

### 3.4 Preliminary Cumulative Risk Assessment Based on Hazard Index

Based on the ATSRD’s guidance manual, if at least two components have HQs > 0.1, further assessment of additivity and interactions are needed (2004a). Therefore, further evaluation of additivity and interactions is necessary for *Mentha canadensis* L., *Lonicera japonica* Thunb., and *Houttuynia cordata* Thunb. used as TCM and food, as well as *Perilla frutescens* (L.) Britton, *Chrysanthemum indicum* L., *Polygonatum odoratum* (Mill.) Druce, and *Zaocys dhumnades* (Cantor) used only as food.

**FIGURE 2:**
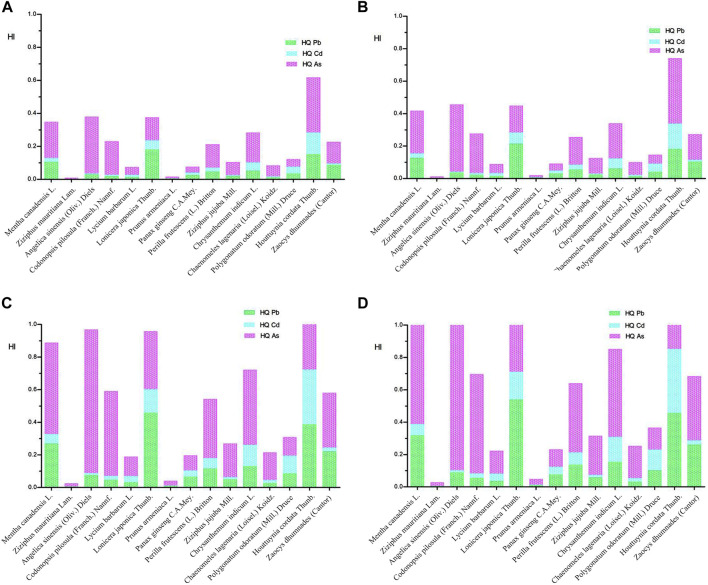
HI of heavy metals in food homologous TCM. **(A)** HI of heavy metals in food homologous TCM used as TCM for male. **(B)** HI of heavy metals in food homologous TCM used as TCM for female. **(C)** HI of heavy metals in food homologous TCM used as food for male. **(D)** HI of heavy metals in food homologous TCM used as food for female.

It was discovered that, if ingested as medicine, the HI values of all assessed food homologous TCM were <1 for both males and females, indicating that the health risks caused by the cumulative exposure of Pb, Cd, and As were acceptable (Figure 2). However, if ingested as food, the HI of *Houttuynia cordata* Thunb. was >1 for both males (1.577) and females (1.861), which revealed that the hazardous health effects associated with Pb, Cd, and As exposure required attention. In addition, the total contribution of Pb, Cd, and As lead to the HIs approaching or surpassing 1 for *Mentha canadensis* L. (1.048), *Angelica sinensis* (Oliv.) Diels (1.143), and *Lonicera japonica* Thunb. (1.129) for females, suggesting that females may experience harmful systemic effects related to co-exposure to Pb, Cd, and As.

Although the HI method usually requires that all chemicals operate under the same mechanism and critical effects, the HI method usually plays the role of a rapid screening tool for pollutants with the same critical target regardless of the mechanism and for contaminants with different target organs. Chemical interactions within the mixtures are not considered by the HI method; therefore, the health hazard may be underestimated when interactions are greater than the additive or be overestimated when interactions are less than the additive. Thus, a more precise cumulative risk assessment is further needed.

### 3.5 Precise Cumulative Risk Assessment

**FIGURE 3:**
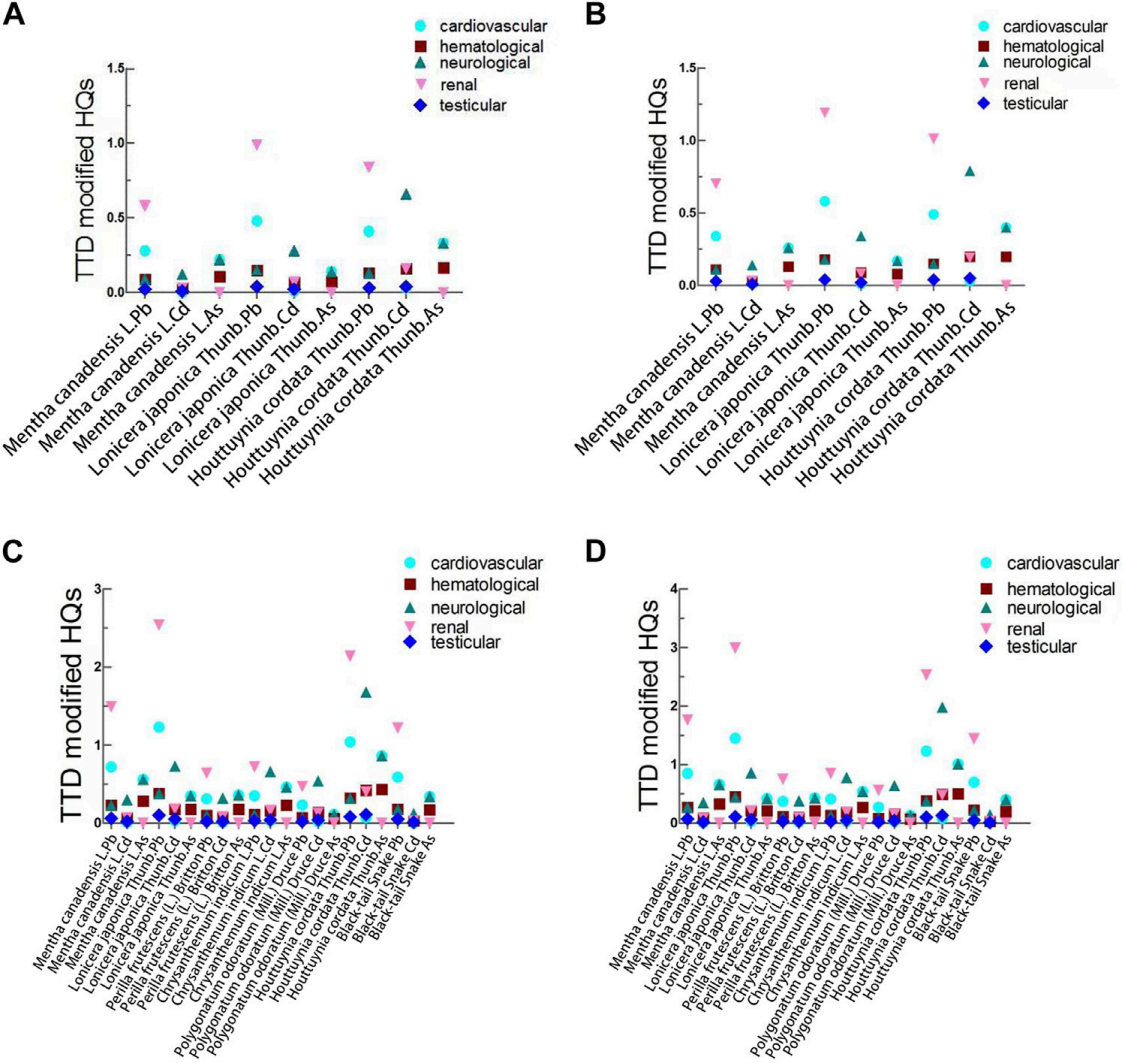
TTD modification of HI. **(A)** TTD modification of HI for food homologous TCM used as TCM for male. **(B)** TTD modification of HI for food homologous TCM used as TCM for female. **(C)** TTD modification of HI for food homologous TCM used as food for male. **(D)** TTD modification of HI for food homologous TCM used as food for female.

The toxic effects of Pb, Cd, and As differ. The cardiovascular system is the most sensitive toxic end-point for Pb. The kidney whose marker is β2-microglobulin is the most sensitive toxic target organ for Cd, and the lung is the most sensitive toxic organ for As. Given the different toxic target organs for Pb, Cd, and As, a more precise cumulative risk assessment is explored in the present study (Figure 3). In the scenario where food homologous TCM were used as TCM, the results of the TTD modification of HI method revealed that the TTD-modified HI values of *Lonicera japonica Thunb.* and *Houttuynia cordata* Thunb. were >1 for both males and females for renal tissue (Table 4). For the neurological system, the TTD-modified HI values of *Houttuynia cordata* Thunb. were >1 for both males and females.

**TABLE 4:** Precise cumulative risk assessment results of MFSV used as TCM based on TTD modification of the HI method.

Types	Target organ	Male				Female			
		TTD-modified HQ of Pb	TTD-modified HQ of Cd	TTD-modified HQ of As	TTD-modified HI	TTD-modified HQ of Pb	TTD-modified HQ of Cd	TTD-modified HQ of As	TTD-modified HI
*Mentha canadensis* L.	Cardiovascular	0.28	0	0.22	0.51	0.34	0.01	0.26	0.61
	Hematological	0.09	0.03	0.11	0.23	0.11	0.03	0.13	0.27
	Neurological	0.09	0.12	0.22	0.42	0.11	0.14	0.26	0.51
	Renal	0.58	0.03	0	0.61	0.70	0.03	0	0.73
	Testicular	0.02	0.01	—	0.03	0.03	0.01	—	0.04
*Lonicera japonica* Thunb.	Cardiovascular	0.48	0.01	0.14	0.63	0.58	0.01	0.17	0.76
	Hematological	0.15	0.07	0.07	0.29	0.18	0.09	0.08	0.35
	Neurological	0.15	0.28	0.14	0.57	0.18	0.34	0.17	0.69
	Renal	0.99	0.07	0	1.06	1.19	0.08	0	1.27
	Testicular	0.04	0.02	—	0.06	0.04	0.02	—	0.07
*Houttuynia cordata* Thunb.	Cardiovascular	0.41	0.03	0.33	0.77	0.49	0.03	0.40	0.92
	Hematological	0.13	0.16	0.17	0.46	0.15	0.20	0.20	0.55
	Neurological	0.13	0.66	0.33	1.12	0.15	0.79	0.40	1.34
	Renal	0.84	0.16	0	1.04	1.01	0.19	0	1.20
	Testicular	0.03	0.04	—	0.08	0.04	0.05	—	0.09

In the scenario where food homologous TCM were used as food, TTD-modified HI values of *Mentha canadensis* L., *Lonicera japonica* Thunb., and *Houttuynia cordata* Thunb. were >1 for both male and female for the cardiovascular system (Table 5). For the hematological system, the TTD-modified HIs of *Houttuynia cordata* Thunb. were >1 for both males and females. For the neurological system, the TTD-modified HI values of *Mentha canadensis* L., *Lonicera japonica* Thunb., *Chrysanthemum indicum* L., and *Houttuynia cordata* Thunb. were >1 for both males and females. For the renal system, the TTD-modified HI values of *Mentha canadensis L*., *Lonicera japonica* Thunb., *Houttuynia cordata* Thunb., and *Zaocys dhumnades* (Cantor) were >1 for both males and females. These results based on the TTD modification of HI method demonstrated that the potential health risks associated with the mixtures of Pb, Cd, and As in *Lonicera japonica* Thunb. and *Houttuynia cordata* Thunb. may exceed the human tolerance dose when they are ingested as both TCM and food for both males and females. Moreover, potential toxic systemic effects related to mixtures of Pb, Cd, and As in *Mentha canadensis* L., *Chrysanthemum indicum* L., and *Zaocys dhumnades* (Cantor) cannot be ignored when they are consumed as food. Thus, further content monitoring and risk assessment of the mixtures of heavy metals in these types of food homologous TCM are of crucial significance.

**TABLE 5:** Precise cumulative risk assessment results of MFSV used as food based on TTD modification of the HI method.

Types	Target organ	Male				Female			
		TTD-modified HQ of Pb	TTD-modified HQ of Cd	TTD-modified HQ of As	TTD-modified HI	TTD-modified HQ of Pb	TTD-modified HQ of Cd	TTD-modified HQ of As	TTD-modified HI
*Mentha canadensis* L.	Cardiovascular	0.72	0.01	0.56	1.30	0.85	0.01	0.66	1.53
	Hematological	0.23	0.07	0.28	0.58	0.27	0.09	0.33	0.68
	Neurological	0.23	0.30	0.56	1.08	0.27	0.35	0.66	1.28
	Renal	1.49	0.07	0	1.56	1.76	0.08	0	1.85
	Testicular	0.06	0.02	—	0.08	0.07	0.02	—	0.09
*Lonicera japonica* Thunb.	Cardiovascular	1.23	0.03	0.35	1.61	1.45	0.03	0.42	1.90
	Hematological	0.38	0.18	0.18	0.74	0.45	0.21	0.21	0.88
	Neurological	0.38	0.73	0.35	1.46	0.45	0.86	0.42	1.73
	Renal	2.54	0.18	0	2.71	2.99	0.21	0	3.20
	Testicular	0.10	0.05	—	0.14	0.11	0.06	—	0.17
*Perilla frutescens* (L.) Britton	Cardiovascular	0.31	0.01	0.36	0.69	0.37	0.02	0.43	0.38
	Hematological	0.10	0.08	0.18	0.36	0.11	0.10	0.21	0.21
	Neurological	0.10	0.32	0.36	0.78	0.11	0.38	0.43	0.49
	Renal	0.64	0.08	0	0.72	0.75	0.09	0	0.85
	Testicular	0.02	0.02	—	0.05	0.03	0.03	—	0.05
*Chrysanthemum indicum* L.	Cardiovascular	0.35	0.03	0.46	0.84	0.41	0.03	0.54	0.99
	Hematological	0.11	0.16	0.23	0.50	0.13	0.19	0.27	0.59
	Neurological	0.11	0.66	0.46	1.23	0.13	0.78	0.54	1.45
	Renal	0.72	0.16	0	0.88	0.85	0.19	0	1.04
	Testicular	0.03	0.04	—	0.07	0.03	0.05	—	0.08
*Polygonatum odoratum* (Mill.) Druce	Cardiovascular	0.23	0.02	0.11	0.37	0.27	0.03	0.13	0.43
	Hematological	0.07	0.14	0.06	0.27	0.08	0.16	0.07	0.31
	Neurological	0.07	0.54	0.11	0.73	0.08	0.64	0.13	0.86
	Renal	0.47	0.13	0	0.61	0.56	0.15	0	0.72
	Testicular	0.02	0.04	—	0.05	0.02	0.04	—	0.06
*Houttuynia cordata* Thunb.	Cardiovascular	1.04	0.07	0.86	1.96	1.23	0.08	1.01	2.32
	Hematological	0.32	0.42	0.43	1.17	0.38	0.49	0.50	1.38
	Neurological	0.32	1.68	0.86	2.86	0.38	1.98	1.01	3.37
	Renal	2.14	0.40	0	2.55	2.53	0.48	0	3.01
	Testicular	0.08	0.11	—	0.19	0.10	0.13	—	0.23
*Zaocys dhumnades* (Cantor)	Cardiovascular	0.59	0.00	0.34	0.93	0.70	0.01	0.40	0.71
	Hematological	0.18	0.03	0.17	0.38	0.22	0.03	0.20	0.25
	Neurological	0.18	0.12	0.34	0.64	0.22	0.14	0.40	0.35
	Renal	1.22	0.03	0	1.25	1.44	0.03	0	1.48
	Testicular	0.05	0.01	—	0.05	0.05	0.01	—	0.06

## 4 Discussion

The general categories of co-exposure toxic actions are additive, more than additive (synergism or potentiation), and less than additive (antagonism, inhibition, or masking), based on the dose-response relationship and interaction mechanism of the individual chemicals (2004a; 2004b). It has been globally recognized that cumulative risk assessment after co-exposure to chemical mixtures is of crucial importance (Scholze et al., 2015; Quijano et al., 2016; Tsatsakis et al., 2016; Zentai et al., 2016); however, it should be noted that there is no universally applicable method for such risk assessments. For instance, benzoapyrene was used as a surrogate chemical by JECFA to evaluate 13 types of carcinogenic polycyclic aromatic hydrocarbons (PAHs) in food using a surrogate approach (WHO, 2006). The surrogate approach was used for the cumulative risk assessment of the mixture, measuring the adverse health effects of multiple substances by the concentration of a single component in the mixture. The surrogate approach assumed that the hazardous effects induced by individual components in the mixture were proportional to the concentration of the “surrogate chemical.” For this approach, the chemical structure and toxicological characteristics of each component should be similar. At least one component has detailed toxicological data, which could be used as the “surrogate chemical.” In addition, the toxic equivalency factor (TEF) (or relative potency factor, RPF) approach is a feasible method for the evaluation of potential health risks associated with exposure to mixtures of polychlorinated dibenzodioxins, polychlorinated dibenzofurans, and coplanar polychlorinated bithenyls (FAO/WHO, 2002). For TEF methods, an “indicator chemical” from a group of chemicals with the same toxicity mechanism needs to be selected. Then, the ratio of the efficiency of each chemical to the efficiency of the indicator chemical should be determined as the correction factor to standardize the exposure of each chemical. Based on the indicator chemical and correction factors, the total exposure and related risks could be calculated. In addition, the physiologically based toxicokinetics (PBTK) model is a well-established approach based on physiology and anatomy. The simulation of absorption, distribution, metabolism, and excretion processes of toxic mixtures in human bodies could be realized by this model under various physiological conditions. The PBTK model offers a highly accurate method for cumulative risk assessment. As an example, Teuschler et al. (2004) explored the application of the PBTK model to evaluate the combined effect of drinking water disinfection by-product mixtures. However, a large number of resources and professional experiences are needed to establish a PBTK model; therefore, this type of approach is rarely used in common risk assessment.

**FIGURE 4:**
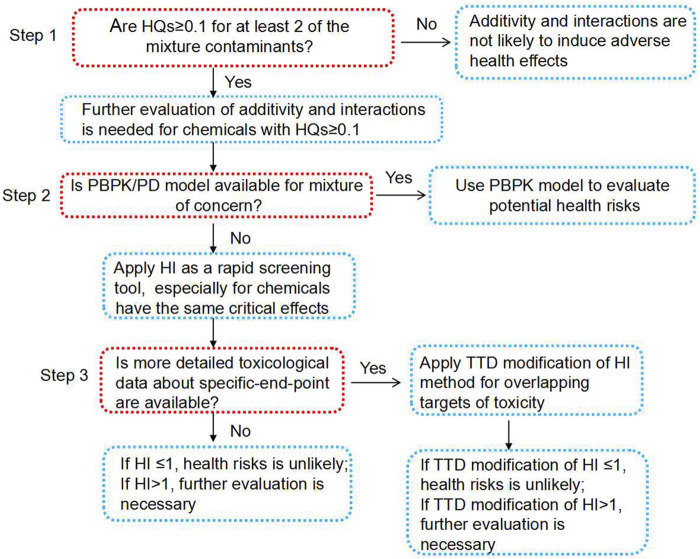
The decision tree for cumulative risk assessment of heavy metals in food homologous TCM.

The chemical structures and toxicity mechanisms of Pb, Cd, and As are entirely different (Gao et al., 2010; Alho et al., 2019; Wang et al., 2020); therefore, neither the surrogate approach nor the TEF approach was suitable for the cumulative risk assessment of these chemicals. To overcome these barriers, the present study developed a three-step integrated tiered strategy called “decision tree” for cumulative risk assessment of heavy metals in food homologous TCM, which was the first of its kind (Figure 4). For step 1, a single-component–based assessment is recommended by using the HQ method, considering the unique characteristics of food homologous TCM used as both TCM and food. If at least two components have HQs higher than 0.1, then further evaluation is necessary, and steps 2 and 3 should be followed. If the requirement of step 1 is not met (which is, “NO” in step 1), the mixture assessment ends with the conclusion “not likely to induce adverse health effects.” If available, step 2 recommends the PBTK models for the evaluation of potential health risks of joint exposure. If not available, the HI method could be applied as a timely screening tool for preliminary accumulative risk assessment by health assessors. In particular, the HI method applies to components with the same critical effects. For step 3, if more detailed toxicological data regarding the specific-end-point are available, the TTD method can be used for overlapping targets of toxicity. Finally, the conclusions at the end of the flowchart can be used to make decisions regarding health hazards and formulate public health actions.

It is noteworthy that food homologous TCM have the characteristics of both medicine and food, and a realistic cumulative risk assessment model was deciphered by the ingestion rates, frequency, and duration of exposure to food homologous TCM acquired by questionnaire data. The safety factor applied to food homologous TCM consumed as TCM and food also differed. This study appears to be the first one to evaluate the cumulative health risks of heavy metals in TCM. The results of our developed decision tree revealed that calculations should not exclude potential cumulative health risks. And policy makers should place more emphasis on the co-exposure of heavy metals in order to make a practical and scientific safety standard for food homologous TCM.

## 5 Conclusion

Using our newly developed three-step decision tree, including hazard quotient (HQ), hazard index (HI), and target-organ toxicity dose (TTD) modification of HI method, we achieved a cumulative risk assessment for co-exposure to Pb, Cd, and As in food homologous TCM based on a real-life-exposure scenario. The results revealed that excluding the HQs of As in *Angelica sinensis* (Oliv.) Diels and *Houttuynia cordata* Thunb. used as food, other HQs of Pb, Cd, or As were lower than 1 for both males and females. However, precise cumulative risk assessment results acquired by the TTD modification of HI method implied that the potential health risks associated with the co-exposure of Pb, Cd, and As in *Lonicera japonica* Thunb. and *Houttuynia cordata* Thunb. ingested as both TCM and food were of concern. Additionally, the cumulative risks of Pb, Cd, and As in *Mentha canadensis* L., *Chrysanthemum indicum* L., and *Zaocys dhumnades* (Cantor) only used as food exceeded the human tolerance dose. Herein, calculations should not rule out potential cumulative health risks and further monitoring of mixed heavy metals in food homologous TCM as well as a cumulative risk assessment are of crucial significance. We hope this study provides novel perspectives for cumulative risk assessment of co-exposure-heavy metals in food homologous TCM, with the main purpose of scientifically using TCM to treat diverse complex diseases in the clinic and improve the public health.

## Data Availability

The original contributions presented in the study are included in the article/Supplementary Material; further inquiries can be directed to the corresponding authors.

## Abbreviations

TCM, traditional Chinese medicine; food homologous TCM, medicine and food homologous varieties; ICP-MS, inductively coupled plasma-mass spectroscopy; EDI, estimated daily intake; HQ, hazard quotient; HI, hazard index; TTD, target-organ toxicity dose; WHO, world health organization; NIFDC, national institutes for food and drug control; NSF, national science foundation; SHBGV, specific health-based guidance values; PAHs, polycyclic aromatic hydrocarbons; TEF, the toxic equivalency factor; RPF, relative potency factor; PBTK, physiologically based toxicokinetics.

## References

[B1] Akbari B. Gharanfoli F. Khayyat M. H. Khashyarmanesh Z. Rezaee R. Karimi G. (2012). Determination of Heavy Metals in Different Honey Brands from Iranian Markets. Food Addit. Contam. Part B Surveill. 5 (2), 105–111. 10.1080/19393210.2012.664173 24779739

[B2] Alho L. O. G. Gebara R. C. Paina K. A. Sarmento H. Melão M. D. G. G. (2019). Responses of Raphidocelis Subcapitata Exposed to Cd and Pb: Mechanisms of Toxicity Assessed by Multiple Endpoints. Ecotoxicol. Environ. Saf. 169, 950–959. 10.1016/j.ecoenv.2018.11.087 30597796

[B3] Arjouni M. Y. Bennouna M. A. El Fels M. A. Romane A. (2015). Assessment of Mineral Elements and Heavy Metals in Leaves of Indigenous Cypress of High Atlas Mountains. Nat. Prod. Res. 29 (8), 764–767. 10.1080/14786419.2014.974052 25421148

[B4] ATSDR (2001). Guidance Manual for the Assessment of Joint Toxic Action of Chemical Mixtures. Atlanta: Agency for Toxic Substances and Disease Registry.

[B5] ATSDR (2004a). Guidance Manual for the Assessment of Joint Toxic Action of Chemical Mixtures. Atlanta: Agency for Toxic Substances and Disease Registry. Availableat: http://www.atsdr.cdc.gov/interactionprofiles/ipga.html .

[B6] ATSDR (2004b). *Interaction Profile for Arsenic, Cadmium, Chromium and Lead* [Online]. Atlanta: Agency for Toxic Substances and Disease Registry. 38498637

[B7] Auyeung T. W. Chang K. K. To C. H. Mak A. Szeto M. L. (2002). Three Patients with Lead Poisoning Following Use of a Chinese Herbal Pill. Hong Kong Med. J. 8 (1), 60–62. 11861997

[B8] Barakat M. A. (2011). New Trends in Removing Heavy Metals from Industrial Wastewater. Arabian J. Chem. 4, 361–377. 10.1016/j.arabjc.2010.07.019

[B9] Bassil M. Daou F. Hassan H. Yamani O. Kharma J. A. Attieh Z. (2018). Lead, Cadmium and Arsenic in Human Milk and Their Socio-Demographic and Lifestyle Determinants in Lebanon. Chemosphere 191, 911–921. 10.1016/j.chemosphere.2017.10.111 29145136

[B10] Bellinger D. C. (2008). Very Low Lead Exposures and Children's Neurodevelopment. Curr. Opin. Pediatr. 20 (2), 172–177. 10.1097/MOP.0b013e3282f4f97b 18332714

[B11] Bolan S. Naidu R. Kunhikrishnan A. Seshadri B. Ok Y. S. Palanisami T. (2016). Speciation and Bioavailability of Lead in Complementary Medicines. Sci. Total Environ. 539, 304–312. 10.1016/j.scitotenv.2015.08.124 26363725

[B12] Bopp S. K. Barouki R. Brack W. Dalla Costa S. Dorne J. C. M. Drakvik P. E. (2018). Current EU Research Activities on Combined Exposure to Multiple Chemicals. Environ. Int. 120, 544–562. 10.1016/j.envint.2018.07.037 30170309 PMC6192826

[B13] Bortey-Sam N. Nakayama S. M. Ikenaka Y. Akoto O. Baidoo E. Mizukawa H. (2015). Health Risk Assessment of Heavy Metals and Metalloid in Drinking Water from Communities Near Gold Mines in Tarkwa, Ghana. Environ. Monit. Assess. 187 (7), 397. 10.1007/s10661-015-4630-3 26038318

[B14] Buettner C. Mukamal K. J. Gardiner P. Davis R. B. Phillips R. S. Mittleman M. A. (2009). Herbal Supplement Use and Blood Lead Levels of United States Adults. J. Gen. Intern Med. 24 (11), 1175–1182. 10.1007/s11606-009-1050-5 19575271 PMC2771230

[B15] Cao L. Du J. Ding W. Jia R. Liu Y. Xu P. (2014). Hepatoprotective and Antioxidant Effects of dietaryAngelica Sinensisextract against Carbon Tetrachloride-Induced Hepatic Injury in Jian Carp (Cyprinus Carpiovar. Jian). Aquac. Res. 47, 1852–1863. 10.1111/are.12643

[B16] Chan K. (2003). Some Aspects of Toxic Contaminants in Herbal Medicines. Chemosphere 52 (9), 1361–1371. 10.1016/s0045-6535(03)00471-5 12867165

[B17] Chen X. Wang K. Wang Z. Gan C. He P. Liang Y. (2014). Effects of Lead and Cadmium Co-exposure on Bone Mineral Density in a Chinese Population. Bone 63, 76–80. 10.1016/j.bone.2014.02.017 24607944

[B18] CMA (2012). Australian Regulatory Guidelines for Complementary Medicines (ARGCM) Part A: General Guidance. Australia: Version 5. Complementary Medicines.

[B19] Council N. R. (1993). Pesticides in the Diets of Infants and Children. Washington DC: National Academy Press. 25144038

[B20] Emenike P. C. Tenebe T. I. Omeje M. Osinubi D. S. (2017). Health Risk Assessment of Heavy Metal Variability in Sachet Water Sold in Ado-Odo Ota, South-Western Nigeria. Environ. Monit. Assess. 189 (9), 480. 10.1007/s10661-017-6180-3 28861723

[B21] EPA (2000). Supplementary Guidance for Conducting Health Risk Assessment of Chemical Mixtures. Italy. Food.EPA/630/R-00/002.Version 5. Codex.

[B22] Evans R. M. Scholze M. Kortenkamp A. (2015). Examining the Feasibility of Mixture Risk Assessment: a Case Study Using a Tiered Approach with Data of 67 Pesticides from the Joint FAO/WHO Meeting on Pesticide Residues (JMPR). Food Chem. Toxicol. 84, 260–269. 10.1016/j.fct.2015.08.015 26344759

[B23] FAO/WHO (2002). Safety Evaluation of Certain Food Additives and Contaminants: Polychlorinated Dibenzodioxins, Polychlorinated Dibenzofurans, and Coplanar Polychlorinated Bithenyls. Geneva: WHO Food Additives Series:.

[B24] Gao X. Schulze D. G. (2010). Chemical and Mineralogical Characterization of Arsenic, Lead, Chromium, and Cadmium in a Metal-Contaminated Histosol. Geoderma 156 (3-4), 278–286. 10.1016/j.geoderma.2010.02.027

[B25] Gündüz O. (2015). Water Quality Perspectives in a Changing World. Water Qual. Expo. Health 7, 1–3. 10.1007/s12403-015-0161-y

[B26] Hughes M. F. (2002). Arsenic Toxicity and Potential Mechanisms of Action. Toxicol. Lett. 133 (1), 1–16. 10.1016/s0378-4274(02)00084-x 12076506

[B27] International N. (2003). Dietary Supplement-Standard 173: Metal Contaminant Accepted Level. Ann Arbor, 3–4.

[B28] Jaishankar M. Tseten T. Anbalagan N. Mathew B. B. Beeregowda K. N. (2014). Toxicity, Mechanism and Health Effects of Some Heavy Metals. Interdiscip. Toxicol. 7 (2), 60–72. 10.2478/intox-2014-0009 26109881 PMC4427717

[B29] Jiang Z. Xu N. Liu B. Zhou L. Wang J. Wang C. (2018). Metal Concentrations and Risk Assessment in Water, Sediment and Economic Fish Species with Various Habitat Preferences and Trophic Guilds from Lake Caizi, Southeast China. Ecotoxicol. Environ. Saf. 157, 1–8. 10.1016/j.ecoenv.2018.03.078 29605640

[B30] Kapaj S. Peterson H. Liber K. Bhattacharya P. (2006). Human Health Effects from Chronic Arsenic Poisoning-Aa Review. J. Environ. Sci. Health A Tox Hazard Subst. Environ. Eng. 41 (10), 2399–2428. 10.1080/10934520600873571 17018421

[B31] Kashyap R. Verma K. S. Uniyal S. K. Bhardwaj S. K. (2018). Geospatial Distribution of Metal(loid)s and Human Health Risk Assessment Due to Intake of Contaminated Groundwater Around an Industrial Hub of Northern India. Environ. Monit. Assess. 190 (3), 136. 10.1007/s10661-018-6525-6 29435679

[B32] Kienzler A. Bopp S. K. van der Linden S. Berggren E. Worth A. (2016). Regulatory Assessment of Chemical Mixtures: Requirements, Current Approaches and Future Perspectives. Regul. Toxicol. Pharmacol. 80, 321–334. 10.1016/j.yrtph.2016.05.020 27211294

[B33] Maghakyan N. Tepanosyan G. Belyaeva O. Sahakyan L. Saghatelyan A. (2017). Assessment of Pollution Levels and Human Health Risk of Heavy Metals in Dust Deposited on Yerevan's Tree Leaves (Armenia). Acta Geochim. 36, 16–26. 10.1007/s11631-016-0122-6

[B34] Mahmood A. Malik R. (2013). Human Health Risk Assessment of Heavy Metals via Consumption of Contaminated Vegetables Collected from Different Irrigation Sources in Lahore. Arab. J. Chem. 7 (1), 91–99.

[B35] Meek M. E. Boobis A. R. Crofton K. M. Heinemeyer G. Raaij M. V. Vickers C. (2011). Risk Assessment of Combined Exposure to Multiple Chemicals: A WHO/IPCS Framework. Regul. Toxicol. Pharmacol. 60, S1–S14. 10.1016/j.yrtph.2011.03.010 21466831

[B36] Nagarajan S. Sivaji K. Krishnaswamy S. Pemiah B. Rajan K. S. Krishnan U. M. (2014). Safety and Toxicity Issues Associated with Lead-Based Traditional Herbo-Metallic Preparations. J. Ethnopharmacol. 151 (1), 1–11. 10.1016/j.jep.2013.10.037 24216165

[B37] Qiu J. (2007). Traditional Medicine: a Culture in the Balance. Nature 448 (7150), 126–128. 10.1038/448126a 17625539

[B38] Quijano L. Yusà V. Font G. Pardo O. (2016). Chronic Cumulative Risk Assessment of the Exposure to Organophosphorus, Carbamate and Pyrethroid and Pyrethrin Pesticides through Fruit and Vegetables Consumption in the Region of Valencia (Spain). Food Chem. Toxicol. 89, 39–46. 10.1016/j.fct.2016.01.004 26774911

[B39] Roba C. Roşu C. Piştea I. Ozunu A. Baciu C. (2016). Heavy Metal Content in Vegetables and Fruits Cultivated in Baia Mare Mining Area (Romania) and Health Risk Assessment. Environ. Sci. Pollut. Res. Int. 23 (7), 6062–6073. 10.1007/s11356-015-4799-6 26062461

[B40] Sanders A. P. Claus Henn B. Wright R. O. (2015). Perinatal and Childhood Exposure to Cadmium, Manganese, and Metal Mixtures and Effects on Cognition and Behavior: A Review of Recent Literature. Curr. Environ. Health Rep. 2 (3), 284–294. 10.1007/s40572-015-0058-8 26231505 PMC4531257

[B41] Singh A. Sharma R. K. Agrawal M. Marshall F. M. (2010). Health Risk Assessment of Heavy Metals via Dietary Intake of Foodstuffs from the Wastewater Irrigated Site of a Dry Tropical Area of India. Food Chem. Toxicol. 48 (2), 611–619. 10.1016/j.fct.2009.11.041 19941927

[B42] Sirot V. Guérin T. Volatier J. L. Leblanc J. C. (2009). Dietary Exposure and Biomarkers of Arsenic in Consumers of Fish and Shellfish from France. Sci. Total Environ. 407 (6), 1875–1885. 10.1016/j.scitotenv.2008.11.050 19103460

[B43] Song C. Liu J. Yao S. Sun J. Hou J. Feng X. (2020). Prevention of New Coronavirus Infection and Screening of Medicinal and Food Homologous TCM. Asia-Pacific Tradit. Med. 16 (11), 18–21.

[B44] Stone R. (2008). Biochemistry. Lifting the Veil on Traditional Chinese Medicine. Science 319 (5864), 709–710. 10.1126/science.319.5864.709 18258866

[B45] Teuschler L. K. Rice G. E. Wilkes C. R. Lipscomb J. C. Power F. W. (2004). A Feasibility Study of Cumulative Risk Assessment Methods for Drinking Water Disinfection By-Product Mixtures. J. Toxicol. Environ. Health A 67 (8-10), 755–777. 10.1080/15287390490428224 15192867

[B46] Tsakiris I. N. Goumenou M. Tzatzarakis M. N. Alegakis A. K. Tsitsimpikou C. Ozcagli E. (2015). Risk Assessment for Children Exposed to DDT Residues in Various Milk Types from the Greek Market. Food Chem. Toxicol. 75, 156–165. 10.1016/j.fct.2014.11.012 25449197

[B47] Tsatsakis A. M. Docea A. O. Tsitsimpikou C. (2016). New Challenges in Risk Assessment of Chemicals when Simulating Real Exposure Scenarios; Simultaneous Multi-Chemicals' Low Dose Exposure. Food Chem. Toxicol. 96, 174–176. 10.1016/j.fct.2016.08.011 27515866

[B48] Wang T. Zheng J. Liu H. Peng Q. Zhou H. Zhang X. (2020). Adsorption Characteristics and Mechanisms of Pb2+ and Cd2+ by a New Agricultural Waste-Caragana Korshinskii Biomass Derived Biochar. Environ. Sci. Pollut. Res. Int. 6, 1–19. 10.1007/s11356-020-11571-9 33191469

[B49] WHO (2006). Evaluation of Certain Food Contaminants. Geneva: WHO technical Report Series. 16708866

[B50] WHO (1997). Food Consumption and Exposure Assessment of Chemicals: Report of FAO/WHO Consultation on Food Consumption and Exposure Assessment of Chemicals. Geneva: WHO.

[B51] WHO (2005). National Policy on Traditional Medicine and Regulation of Herbal Medicines-Report of a WHO Global Survey. Geneva: WHO, 168.

[B52] Yue S. J. Liu J. Feng W. W. Zhang F. L. Chen J. X. Xin L. T. (2017). System Pharmacology-Based Dissection of the Synergistic Mechanism of Huangqi and Huanglian for Diabetes Mellitus. Front. Pharmacol. 8, 694. 10.3389/fphar.2017.00694 29051733 PMC5633780

[B53] Zentai A. Szabó I. J. Kerekes K. Ambrus Á. (2016). Risk Assessment of the Cumulative Acute Exposure of Hungarian Population to Organophosphorus Pesticide Residues with Regard to Consumers of Plant Based Foods. Food Chem. Toxicol. 89, 67–72. 10.1016/j.fct.2016.01.016 26807885

[B54] Zhang W. L. Du Y. Zhai M. M. Shang Q. (2014). Cadmium Exposure and its Health Effects: a 19-year Follow-Up Study of a Polluted Area in China. Sci. Total Environ. 470-471, 224–228. 10.1016/j.scitotenv.2013.09.070 24140693

[B55] Zuo T. T. Jin H. Y. Zhang L. Liu Y. L. Nie J. Chen B. L. (2020a). Innovative Health Risk Assessment of Heavy Metals in Chinese Herbal Medicines Based on Extensive Data. Pharmacol. Res. 159, 104987. 10.1016/j.phrs.2020.104987 32512044

[B56] Zuo T. T. Qu H. R. Jin H. Y. Zhang L. Luo F. Y. Yu K. Z. (2020b). Innovative Health Risk Assessments of Heavy Metals Based on Bioaccessibility Due to the Consumption of Traditional Animal Medicines. Environ. Sci. Pollut. Res. Int. 27 (18), 22593–22603. 10.1007/s11356-020-08769-2 32319064

