# Supplementary figures and images for: Novel Integrated Tiered Cumulative Risk Assessment of Heavy Metals in Food Homologous Traditional Chinese Medicine Based on a Real-Life-Exposure Scenario
Source: Front Pharmacol. 2022 Jun 24;13:908986. doi: 10.3389/fphar.2022.908986 (PMC9268897; doi:10.3389/fphar.2022.908986)

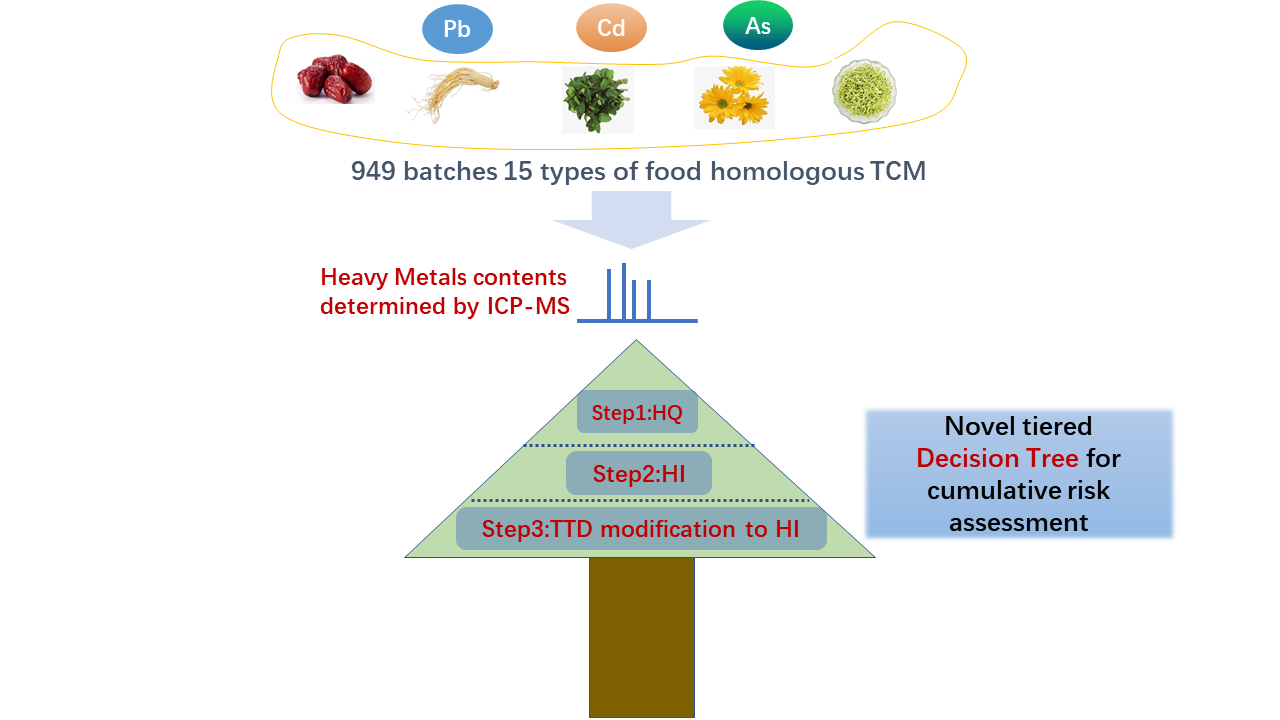

Supplement: Supplementary file 1 [file Image1.PNG]
